# Single-cell mapping of DNA G-quadruplex structures in human cancer cells

**DOI:** 10.1038/s41598-021-02943-3

**Published:** 2021-12-08

**Authors:** Winnie W. I. Hui, Angela Simeone, Katherine G. Zyner, David Tannahill, Shankar Balasubramanian

**Affiliations:** 1 Cancer Research UK Cambridge Institute, Li Ka Shing Centre, University of Cambridge, Robinson Way, Cambridge, CB2 0RE UK; 2 Yusuf Hamied Department of Chemistry, University of Cambridge, Cambridge, CB2 1EW UK; 3 School of Clinical Medicine, University of Cambridge, Cambridge, CB2 0SP UK

**Keywords:** DNA, Next-generation sequencing, Genomics, Biological techniques, Epigenetics

## Abstract

G-quadruplexes (G4s) are four-stranded DNA secondary structures that form in guanine-rich regions of the genome. G4s have important roles in transcription and replication and have been implicated in genome instability and cancer. Thus far most work has profiled the G4 landscape in an ensemble of cell populations, therefore it is critical to explore the structure–function relationship of G4s in individual cells to enable detailed mechanistic insights into G4 function. With standard ChIP-seq methods it has not been possible to determine if G4 formation at a given genomic locus is variable between individual cells across a population. For the first time, we demonstrate the mapping of a DNA secondary structure at single-cell resolution. We have adapted single-nuclei (sn) CUT&Tag to allow the detection of G4s in single cells of human cancer cell lines. With snG4-CUT&Tag, we can distinguish cellular identity from a mixed cell-type population solely based on G4 features within individual cells. Our methodology now enables genomic investigations on cell-to-cell variation of a DNA secondary structure that were previously not possible.

## Introduction

Certain guanine-rich nucleic sequences can fold into four-stranded DNA secondary structures called G-quadruplexes (G4s), due to the ability of guanine to self-associate through Hoogsteen hydrogen bonding. Our invention of chromatin immunoprecipitation of G4s using a structure-specific antibody coupled with high-throughput sequencing (G4-ChIP-seq) allowed the genome-wide mapping of G4s^1^, and such studies support their involvement in vital cellular processes such as transcription, replication and genome stability^2^. Furthermore, using patient-derived tumour xenografts we showed that G4 signatures uncover transcriptional features and genome alterations in breast cancer, revealing predictable responses to G4 stabilising ligand treatment^3^. However, the averaging of ChIP-seq signals across a cellular population limits our understanding of the differences in chromatin structures between individual cells.

Single-cell (sc) methods^4–9^ have been developed to discern molecular variations and cell identity within cellular populations, which have enhanced our understanding of biology such as cell-type specification^10^ and intratumour heterogeneity^11^. Given G4s are fundamental features of genomic DNA, a method for profiling of G4s at the single-cell level would reveal the importance of G4s to cellular heterogeneity and how genome regulatory features relate to G4s in individual cells within a cellular population. Previously, imaging^12–14^ and cytometry^15^ studies of single cells showed that G4 formation is dynamic and cell-cycle dependent but precise genomic location and sequence context of G4s in individual cells were elusive. Here, we have adapted Cleavage Under Targets and Tagmentation (CUT&Tag)^6–8^ for G4s and applied it to map G4 locations at single-cell level, for the first time.

## Results and discussion

### CUT&Tag robustly maps G4s

**Figure 1:**
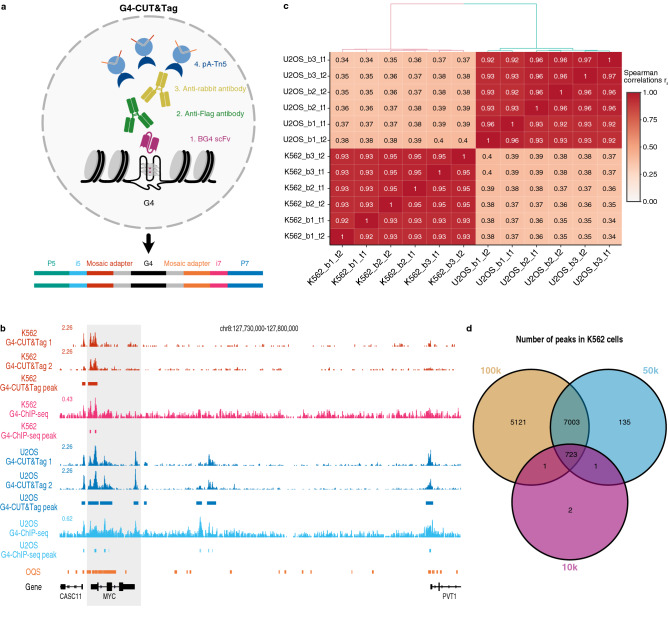
Characterisation of bulk G4-CUT&Tag. (**a**) Schematic diagram of G4-CUT&Tag workflow. Fixed permeabilised cells are incubated with the G4 structure-specific BG4 single-chain variable fragment (scFv), followed the secondary antibodies, and then adapter-loaded proteinA-Tn5 to enable the integration of adapters at G4 target loci for next-generation sequencing library preparation. (**b**) Example genome browser view of G4-CUT&Tag signals and G4 peaks called obtained from 100,000 K562 (red) and 100,000 U2OS (blue) cells with comparisons to published G4-ChIP-seq data^21^ (pink, light blue respectively), and sites that fold into G4 structures in vitro (called observed quadruplex sequences OQs, orange)^20^ Light grey shading highlights G4 peaks in the MYC locus. Gene annotations are shown in black below. (**c**) Hierarchical clustering of the Spearman correlation matrix for G4-CUT&Tag replicates. G4-CUT&Tag was performed on three biological replicates (b1, b2, b3) with two technical replicates (t1, t2) for 100,000 K562 and 100,000 U2OS cells. Spearman correlations between samples were computed at G4 peaks on read coverage normalised to library size. (**d**) Venn diagram showing the number of G4 peaks and their overlap from 100,000 (100 k), 50,000 (50 k) and 10,000 (10 k) K562 cells.

First, we established G4-CUT&Tag using human chronic myelogenous leukaemia (K562) and osteosarcoma (U2OS) cell lines to demonstrate applicability of the method for both suspension and adherent cell lines. We employed buffers using potassium salts, rather than sodium, to mimic intracellular ionic conditions, since cation coordination with K^+^ can influence G4 stability^16^. 100,000 fixed permeabilised cells^17^ were incubated with a FLAG-tagged G4 structure-specific single-chain variable fragment (BG4)^12^. Next, a rabbit anti-FLAG secondary antibody followed by an anti-rabbit tertiary antibody were added followed by an adapter-loaded protein A-Tn5 transposome (with FLAG epitope removed, see Supplementary Information). Tagmentation was then performed with magnesium-induced activation of Tn5 to insert mosaic-end adapters at target sites. Tagmented fragments were subsequently enriched by PCR from purified genomic DNA, pooled and sequenced (Fig. 1a).

G4 peaks were defined as local enrichment in G4-CUT&Tag data using the SEACR peak caller^18,19^ (see Supplementary Information), and showed high overlap (> 60%) with G4 structures identified in vitro in purified human genomic DNA (referred to as observed quadruplex sequences, OQs)^20^. When compared to G4-ChIP-seq data, > 97% of G4-ChIP-seq peaks were seen in G4-CUT&Tag data in both cell lines^21,22^ (Fig. 1b and Supplementary Table S1). We observed low background, which is a characteristic of CUT&Tag data^6^, with the average fraction of reads in G4 peaks being above 40% (Fig. 1b and Supplementary Table S1). Technical and biological replicates showed high reproducibility (Spearman’s rank correlation coefficient, r_s_ > 0.9) within the same cell line (Fig. 1c). Compared to samples incubated with negative-control IgG antibodies, G4-CUT&Tag libraries had a higher library concentration, supporting G4 enrichment via antibody-directed tagmentation (Supplementary Fig. S1). We observed G4s unique to each cell line with 6161 (34%) of K562 and 23,072 (65%) of U2OS G4 peaks. Taken together, our data shows that G4-CUT&Tag is a robust and reproducible technique to probe G4 structures at the genomic scale. Compared to conventional G4-ChIP-seq^23^, G4-CUT&Tag method does not require sonication and requires a 100-fold less cellular input (100,000 cells for G4-CUT&Tag and 1 × 10^7^ cells for chromatin preparation in G4-ChIP-seq^23^).

To profile G4s in situations where cell number is limiting, we performed G4-CUT&Tag on 50,000 and 10,000 K562 cells. While the total number of G4 peaks was lower, both cell profiles represented an almost complete subset of the 100,000-cell G4-CUT&Tag landscape (98% for 50,000 and 99% for 10,000 cells respectively), and had high specificity for G4s (> 85% overlap with OQs) (Fig. 1d and Supplementary Table S1).

### G4 mapping at the single-cell level

**Figure 2:**
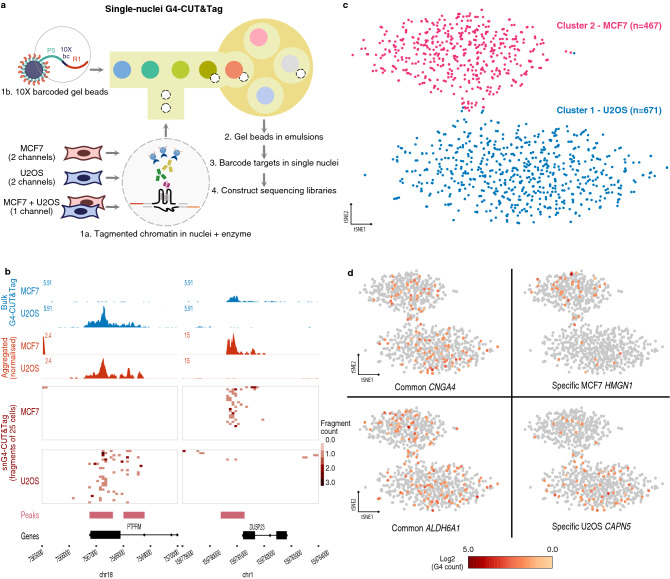
Characterisation of single-nuclei G4-CUT&Tag. (**a**) Schematic diagram of single-nuclei G4 CUT&Tag (snG4-CUT&Tag) workflow. In fixed nuclei, G4-CUT&Tag is used to integrate adapters at G4 sites. Single intact nuclei are then partitioned with barcoded gel beads to index tagmented fragments from individual nuclei using a 10X Genomics Chromium platform with Next GEM Single Cell ATAC Reagents Kits (see “Methods”). (**b**) Two example G4 landscapes in the human genome generated by G4-CUT&Tag. Blue genomic tracks: normalised read coverages of bulk G4-CUT&Tag libraries (50,000 MCF7 cells and 100,000 U2OS cells); red genomic tracks: normalised signals from aggregated snG4-CUT&Tag libraries; dark red tiles: top 50 single nuclei based on the total number of fragments in the genomic region; pink: Cell Ranger peaks called; black: gene annotations. (**c**) Clustering of snG4-CUT&Tag data reveals two groups in mixed U2OS and MCF7 sample. t-SNE plot showing dimensionality reduced snG4-CUT&Tag data from mixed cell lines with 671 imputed U2OS (blue) and 467 imputed MCF7 (red) cells. (**d**) Graphical representation of G4 distribution across promoter G4 peaks in single cells. As in (**c**) for each t-SNE plot, the top cluster represents individual MCF7 cells while the bottom cluster represents individual U2OS cells. Each dot shows example data covering single U2OS or MCF7 cells for the indicated genes. Grey colouring indicates that no G4 is detected in that cell for Cell Ranger-called promoter peak(s) (1000 bases upstream or 100 bases downstream from the TSS) of the specified genes while orange to red shading quantifies the number of G4s (i.e. pA-Tn5 cut sites) detected in a single cell at the promoter peak(s) of the specified gene. Two example genes (*CNGA4* and *ALDH6A1*) are shown where the G4 promoter peaks are observed in common in the two cell lines, and two example genes (*CAPN5* and *HMGN1*) are shown in which the with G4 promoter peaks specific to only one cell line.

We next sought to deploy G4-CUT&Tag to profile G4s at the single-cell level using single nuclei (sn). We prepared two biological replicates of fixed nuclei from MCF7 breast adenocarcinoma and U2OS osteosarcoma adherent cell lines, as this enabled parallel processing under the same conditions thus allowing for a fair comparison of data obtained from different cell types. Each set of nuclei was subjected to G4-CUT&Tag followed by single nuclei partitioning and barcoding using a 10X Genomics microfluidic platform (Fig. 2a). Using 10X Genomics Cell Ranger software, we obtained G4 profiles of an average of 593 MCF7 and 2,071 U2OS cells with a median of 739 and 939 unique fragments per cell, respectively (Supplementary Table S2). Compared to other studies, albeit in different cell types, we observed a greater number of median unique fragments per cell (~ 650–1200) compared to scCUT&Tag for transcription factors (< 300)^7^ but similar to that of H3K27me3 (~ 400–6000)^8^. The tracks of normalised read coverage from aggregated single cells correlate well (r_s_ > 0.8, Supplementary Fig. S2) with that of bulk G4-CUT&Tag from ~ 10,000 cells (Fig. 2b), confirming that snG4-CUT&Tag data recapitulates the observations from bulk G4-CUT&Tag. Also, there is high reproducibility between biological replicates of snG4-CUT&Tag experiment (r_s_ > 0.7, Supplementary Fig. S2). Single-cell G4 peaks (local enrichments called by Cell Ranger from aggregated single-cell profiles), showed good overlap with OQs and ensemble G4-ChIP-seq maps (Supplementary Table S2). These observations confirm the veracity of snG4-CUT&Tag to probe G4 landscapes in individual cells with high specificity and reproducibility.

### snG4-CUT&Tag readily resolves cell identity in a mixed cell population

We next explored whether snG4-CUT&Tag could distinguish cell types based on single-cell G4 profiles. We mixed tagmented MCF7 and U2OS nuclei in a 1:1 ratio and subjected them to single nuclei partitioning and sequencing. Two distinct clusters of cells (n = 467 and 671) were observed after dimensionality reduction using latent sematic indexing followed by graph-based clustering and visualization via t-SNE projection, using the 10X Genomics Cell Ranger ATAC pipeline (Fig. 2c). For the aggregated single-cell profiles of each cluster, through hierarchical clustering with aforementioned bulk G4-CUT&Tag and snG4-CUT&Tag dataset, we imputed the cell identity of the two clusters and successfully resolved them as either MCF7 or U2OS cells (Supplementary Fig. S2).

As many G4s are found in promoters of active and amplified genes^3^, we next investigated if we could observe any difference between the two clusters based on promoter G4 peaks called by Cell Ranger. For the MCF7 cluster, we found that 28% of the top 50 differentially enriched promoter G4 peaks were associated with genes, such as *BCAS1* and *BRIP1*, showing increased copy number (log2(relative to ploidy + 1) > 1.5, Supplementary Table S3)^24^. This result from unsupervised single-cell analysis is consistent with earlier observations of G4s in amplified regions in breast cancers^3^.

### snG4-CUT&Tag reveals cell-to-cell variation of G4 formation

We further investigated the cell-to-cell variation of G4 formation within each cluster by quantifying the number of cells displaying a G4 structure at a given genomic locus within each cell type. We counted the number of supporting cells, defined as a cell having at least one unique read identified per G4 peak called by MACS2, for both U2OS and MCF7 clusters independently (Supplementary Fig. S3a,b). We found that G4 peaks with a higher number of supporting cells tended to be common to both cell lines, whereas G4 peaks with fewer supporting cells tend to be cell line-specific (Supplementary Fig. S3a,c). However, data from many more cells may be needed to further validate this observation. Nevertheless, our snG4-CUT&Tag data showed variable frequency of G4s at certain loci within a cell population (Fig. 2d). Such findings provide a snapshot the co-occurrence of G4s in individual cells thus providing information previously unobtainable by ensemble measurements.

### Implications of snG4-CUT&Tag findings

This study is the first example that we are aware of that has mapped DNA secondary structure at single-cell level. The separation of cell types by snG4-CUT&Tag demonstrates that the pattern of G4s reflect cellular identity right down to the single-cell level. We observe gene-specific incidence of G4s in sub-populations of cells, previously inaccessible by ensemble measurements. In future, snG4-CUT&Tag will be applied to a variety of sample types and cell states, to provide more insights into the roles of DNA secondary structures in biology. Potential applications of the approach include the determination of cellular heterogeneity in tumour cell populations and also biological changes during developmental processes and disease states. Moreover, we envisage that the incorporation of G4s into single-cell multimodal omics approaches will provide a more complete understanding of how chromatin structure and gene expression are regulated at individual loci^25,26^.

## Methods

Procedures to prepare BG4 scFv antibody and transposome and detailed experimental and analysis protocol of bulk G4-CUT&Tag are described in the Supplementary Information.

### Single-nuclei G4-CUT&Tag

The single-nuclei (sn) CUT&Tag protocols described in Bartosovic et al*.*^7^ and Wu et al*.*^8^ were modified to allow profiling of G4 DNA secondary structures in single nuclei. To prepare fixed nuclei, cells were harvested, counted and centrifuged for 5 min at 250×*g* at room temperature. Aliquots of cells (1,000,000 cells per reaction) were resuspended in 0.1% formaldehyde in PBS for 2 min at room temperature and quenched with glycine to a final concentration of 0.075 M. Fixed cells were centrifuged at 1300×*g* at 4 °C for 4 min and resuspended in 1 mL NE1 buffer (20 mM HEPES pH7.5, 10 mM KCl, 0.5 mM spermidine, 0.1% Triton X-100, 20% glycerol in nuclease-free water with a Roche Complete Protease Inhibitor EDTA-Free tablet) for 10 min on ice to release nuclei^27^. Nuclei were harvested by centrifugation at 1300×*g* at 4 °C for 4 min, washed once with 1 mL PBS and resuspended in 500 µL 1% BSA antibody buffer (2 mM EDTA, 1% BSA, 0.05% digitonin, 20 mM HEPES pH7.5, 150 mM KCl, 0.5 mM spermidine in nuclease-free water with a Roche Complete Protease Inhibitor EDTA-free tablet). Nuclei were checked for integrity and counted by staining with trypan blue using hemocytometer. The following wash steps were performed using centrifugation at 4 °C for 3 min at 600×*g* in a swinging bucket rotor, and then at 4 °C for 3 min at 300×*g* following the third antibody incubation step. 500,000 fixed nuclei in 1% BSA antibody buffer were transferred to 0.2 mL Lo-retention tubes (Axygen, AXY2034) and washed twice with 200 µL 1% BSA antibody buffer. Nuclei were resuspended in 50 µL 1% BSA antibody buffer and incubated at 25 °C for 1 h at 600 rpm. 2 µL of 5.4 µM BG4 antibody was added and incubated in the cold room overnight at 600 rpm. Nuclei were then washed twice with 100 µL dig-wash buffer (0.05% digitonin, 20 mM HEPES pH 7.5, 150 mM KCl, 0.5 mM spermidine in nuclease-free water with a Roche Complete Protease Inhibitor EDTA-free tablet) and resuspended in 50 µL dig-wash buffer. 2 µL of rabbit anti-FLAG antibody (Cell Signaling Technology Cat# 2368, RRID:AB_2217020) were added and samples incubated at 25 °C for 1 h at 600 rpm. Nuclei were washed twice with 100 µL dig-wash buffer and resuspended in 50 µL dig-wash buffer before adding 0.5 µL of anti-rabbit antibody (Antibodies-Online Cat# ABIN101961, RRID:AB_10775589) and incubating at 25 °C for 1 h at 600 rpm. Nuclei were then washed three times with 100 µL Dig-300 buffer (0.01% digitonin, 20 mM HEPES pH7.5, 300 mM KCl, 0.5 mM spermidine in nuclease-free water with a Roche Complete Protease Inhibitor EDTA-free tablet) and incubated in 1:250 dilution of pA-Tn5 in 50 µL Dig-300 buffer at 25 °C for 1 h at 600 rpm. Nuclei were washed three times with 100 µL Dig-300 buffer and tagmentation activated by incubation in 100 µL tagmentation buffer (10 mM MgCl_2_ in Dig-300 buffer) at 37 °C for 1 h in thermocycler and mixed every 10 min. After tagmentation, BSA was added to a final concentration of 1% and samples centrifuged at 4 °C for 3 min at 600×*g*. Nuclei were washed twice with 1× diluted nuclei buffer (10X Genomics, 2000207) with 1% BSA by centrifugation at 4 °C for 3 min at 600×*g* and resuspended in 25 µL 1× diluted nuclei buffer on ice. Nuclei were again counted and checked for integrity using trypan blue staining and a hemocytometer. To barcode tagmented regions in single nuclei, the Chromium Next GEM Single Cell ATAC Reagents Kit v1.1 (10X Genomics, 1000175) was used according to the manufacturer with the following modifications. In step 1, 5 µL of nuclei suspension (at about 2500 nuclei/µL) in 1X diluted nuclei buffer (10X Genomics, 2000207) were mixed with 7 µL of ATAC buffer (10X Genomics, 2000193) and 3 µL of low TE but the isothermal incubation was omitted. All the remaining steps from step 2 were performed as per the manufacturer’s instructions. Libraries were sequenced on a NextSeq 500 sequencer (Illumina) with a paired-end format of 50 bp × 2 using the High Output kit (Illumina, FC-404-2002).

### Single-nuclei G4-CUT&Tag data processing

#### Fastq alignment

Two libraries for each cell line (MCF7 and U2OS) and one library of mixed MCF7 and U2OS cells (referred to as “real” mixed) were prepared and sequenced on an Illumina NextSeq 500 instrument. After quality control with FastQC, fastq files were processed using the count function of the 10X Genomics Cell Ranger ATAC software package (cellranger-atac-2.0.0). Reads were aligned to the hg38 genome reference. Artificially mixed fastq files were also generated by mixing one replicate of MCF7 and one replicate of U2OS that were sequenced together. Cell Ranger ATAC identified peaks on bulk data and identified cells (MCF7:343 and 843 cells; U2OS: 2089 and 2053 cells) with median high-quality fragments per cell ranging between 652 and 1226 (see Supplementary Table S2). For the mixed cell samples (real and artificial), Cell Ranger ATAC performed dimensionality reduction; subsequent clustering (graphclust) identified two distinct clusters for each case. The outcome of the Cell Ranger ATAC clustering was also recapitulated using Seurat and Signac R packages. H5 files, fragment files and singlecells files generated by Cell Ranger ATAC were used for analysis in R.

#### Single cell and bulk comparisons

With the output from the graphClust clustering step (where barcodes are paired to each of identified clusters) and the possorted bam file (position-sorted barcode-corrected aligned reads), sinto (sinto filterbarcodes) was used to filter reads and generate a bam file for each cluster with reads belonging to the clustered barcodes. The resulting bam files were used to generate signal tracks (bigwig) scaled to the total number of reads and to identify peaks using macs2 (-p 1e−5 -nomodel -extsize 147); peaks were additional filtered by qvalue (− log10(qvalue) ≥ 800).

#### Single-cell aggregate and cluster cell-identity analysis

To determine cell identity of each single-cell cluster, an unsupervised clustering analysis on the G4 signal was performed. First, the consensus G4 regions observed in U2OS and MCF7 in bulk G4-CUT&Tag were merged to create a reference genomic set. Next, the signals for each single-cell aggregate cluster bam (2 clusters for each mixed case, in total 4 clusters), all individual single cells libraries (2 replicate for each cell lines, in total 4 libraries) and bulk samples (independently sequenced, 2 replicates for each cell lines, in total 4 libraries) were estimated for the previously defined reference set. The G4 signal was calculated as pileups at the genomic loci considered (39,040 regions) and normalised by total number of reads obtaining reads per million mapped reads (RPMs). RPMs were used to compute pairwise Spearman correlations across all libraries. A matrix of 12 columns (libraries) and 39,040 rows (G4 loci) was subjected to z-score transformation and values above the 75th quantile were fixed to the 75th quantile value. The resulting matrix was rescaled between 0 and 1, where 0 indicates low and 1 high G4 signal, and clustered by CLARA (Clustering Large Application, an extension of the K-medoid PAM, K = 4).

#### G4 loci supporting cells

Barcodes of the clustered cells (graphClust output) were used with the fragment file to generate a file of fragment file per cluster. Next, for each individual cluster MACS2 peaks obtained from the aggregated bam data were compared to corresponding cluster fragment file (bedtools intersect). G4 regions were annotated by reporting how many barcodes showed one or more fragments that overlap the region (groupBy -g 1,2,3,4 -c 14 -o count_distinct) resulting in one bed file for each cluster containing the G4 coordinates and the number of barcodes (cells) supporting each locus. The two bam files were intersected to generate a list of overlapping regions and the set of regions specific for each cluster resulting in three separate beds. For each group, the global distribution of the numbers-of-cell support across the G4 loci and the top 25% supported loci were extracted and used for the subsequent characterisation.

#### Enriched promoter G4 peaks and copy number status

With the use of the Significant Features Tool (10X Genomics Loupe Browser 5.0.1), the number of cut sites at Cell Ranger peaks at promoter (1000 bases upstream or 100 bases downstream from the TSS) between U2OS and MCF7 clusters in the mixed sample was computed and the top 50 differentially enriched promoter peaks (absolute log2FC > 0.6 and p-value < 0.05) was identified. The list was compared against DepMap gene level copy number data (log2(relative to ploidy + 1))^24^ of each cell line.

## Supplementary Information

Supplementary Information 1.

Supplementary Table S1.

Supplementary Table S2.

Supplementary Table S3.

## Data Availability

The data discussed in this publication have been deposited in NCBI's Gene Expression Omnibus and are accessible through GEO Series accession number GSE181373 (https://www.ncbi.nlm.nih.gov/geo/query/acc.cgi?acc=GSE181373).

## Code availability

Scripts and general codes for G4-CUT&Tag data analysis are available at https://github.com/sblab-bioinformatics/BG4_CUT_and_Tag_sc.
