# Publisher Correction: Single-cell mapping of DNA G-quadruplex structures in human cancer cells

**DOI:** 10.1038/s41598-022-05047-8

**Published:** 2022-01-12

**Authors:** Winnie W. I. Hui, Angela Simeone, Katherine G. Zyner, David Tannahill, Shankar Balasubramanian

**Affiliations:** 1 Cancer Research UK Cambridge Institute, Li Ka Shing Centre, University of Cambridge, Robinson Way, Cambridge, CB2 0RE UK; 2 Yusuf Hamied Department of Chemistry, University of Cambridge, Cambridge, CB2 1EW UK; 3 School of Clinical Medicine, University of Cambridge, Cambridge, CB2 0SP UK

Correction to: *Scientific Reports* 10.1038/s41598-021-02943-3, published online 08 December 2021

The original version of this Article contained an error in the link in the Code Availability section where,

“Scripts and general codes for G4-CUT&Tag data analysis are available at https://github.com/sblabbioinformatics/BG4_CUT_and_Tag_sc.”

now reads:

“Scripts and general codes for G4-CUT&Tag data analysis are available at https://github.com/sblab-bioinformatics/BG4_CUT_and_Tag_sc.”

The original Article has been corrected.

