# Supplementary material for: Single-cell mapping of DNA G-quadruplex structures in human cancer cells
Source: Sci Rep. 2021 Dec 8;11:23641. doi: 10.1038/s41598-021-02943-3 (PMC8654944; doi:10.1038/s41598-021-02943-3)
Supplement: Supplementary file 1 — Supplementary Information 1. [file 41598_2021_2943_MOESM1_ESM.pdf]

Single-cell mapping of DNA G-quadruplex structures in human cancer cells

Winnie W.I. Hui[1], Angela Simeone[1], Katherine G. Zyner[1], David Tannahill[1], Shankar Balasubramanian[1,2,3*]

[1] Cancer Research UK Cambridge Institute, University of Cambridge, Li Ka Shing Centre, Robinson Way, Cambridge CB2 0RE, UK.

[2] Yusuf Hamied Department of Chemistry, University of Cambridge, Cambridge, CB2 1EW, UK

[3] School of Clinical Medicine, University of Cambridge, Cambridge, CB2 0SP, UK

[*]e-mail: sb10031@cam.ac.uk

Table of contents

List of Supplementary Tables **2**

Supplementary Methods **3**

Cell Culture 3

BG4 scFv antibody generation 3

Transposome preparation. 4

Bulk G4-CUT&Tag 7

Bulk G4-CUT&Tag data processing. 9

Supplementary Figures **11**

Supplementary Fig. S1 | Comparison of G4-CUT&Tag libraries relative to negative controls. 11

Supplementary Fig. S2 | Single-nuclei G4-CUT&Tag can identify separate cell types in a mixed population. 12

Supplementary Fig. S3 | Subtypes of G4s in snG4-CUT&Tag data of mixed U2OS and MCF7 cells. 13

References **14**

List of Supplementary Tables

Supplementary Table S1 | Basic statistics of bulk G4-CUT&Tag data

Basic alignment and peak statistics of individual bulk G4-CUT&Tag libraries and consensus regions called from replicates of each cell line (K562, U2OS and MCF7). This table shows the number of mapped reads, duplication rate, number of peaks called by SEACR and their overlapping with reference dataset, and the fraction of reads in peak (FRiP).

Supplementary Table S2 | Basic statistics of single-nuclei G4-CUT&Tag data

Basic alignment, cell calling and peak statistics of individual snG4-CUT&Tag libraries. This table includes the number of cells identified by Cell Ranger, number of fragments per cell, number of Cell Ranger peaks and their overlapping with reference dataset, and sequenced read statistics.

Supplementary Table S3 | Top 50 differentially enriched promoter G4 peaks

This table includes a list of top 50 genes with differentially enriched promoter G4 peaks between two clusters from snG4-CUT&Tag data in the mixed MCF7+U2OS sample obtained from 10X Genomics Loupe Browser 5.0.1 and their relative copy number from DepMap dataset for each cell line.

Supplementary Methods

Cell Culture

K562 cells (RRID:CVCL_0004) were cultured in RPMI 1640 (Gibco, 21875034) supplemented with 10% heat-inactivated fetal bovine serum (FBS, Gibco, A3840401). U2OS cells (RRID:CVCL_0042) were cultured in DMEM (Gibco, 41966029), 10% FBS. MCF7 cells (RRID:CVCL_0031) were cultured in DMEM, 10% FBS, 1% Penicillin-Streptomycin (Gibco, 15070063). Cell lines were maintained at 37 °C with 5% CO_2. All cell lines were authenticated using short tandem repeat (STR) DNA profiling and tested negative for mycoplasma contamination by qPCR.

BG4 scFv antibody generation

The BG4 scFv antibody was expressed, purified and validated essentially as described previously[1,2]. pSANG10-3F-BG4 (Addgene plasmid #55756) was transformed to BL21 (DE3) competent *E. coli* (NEB, C2527) and inoculated in 2X YT medium (Formedium, YDB0102) with 50µg/mL kanamycin and 2% glucose and incubated at 30°C overnight at 200rpm. 200µL of the overnight culture was grown in 100mL auto-induction media ZYM5052 (2mM $MgSO_4$, 0.2X metal mix, 1X 5052, 1X M and 50µg/mL kanamycin in ZY medium (10g N-Z amine AS (Sigma-Aldrich, N4517), 5g yeast extract (ForMedium, YEA03) in 1 litre water) at 37°C for 6h at 250rpm then incubated overnight at 25°C at 280rpm. Cells were harvested by centrifugation at 4°C for 30min at 4,000xg. Cell pellets were resuspended in 8mL ice-cold TES (50mM Tris-HCl pH8.0, 1mM EDTA pH 8.0, 20% sucrose in water and filtered) and left on ice for 10min. 12mL of 1:5 diluted ice-cold TES (1:5 TES diluted in 300mL MilliQ water with 28µL benzonase (Sigma, E1014-5KU) and 2mM $MgSO_4$) was added and left on ice for 15min. The cell suspension was centrifuged at 4°C for 10min at

8,000xg and the supernatant filtered through a 0.45μm protein low-bind membrane (Millipore, SLHV033RS). The supernatant was incubated with PBS-equilibrated HIS-select Nickel Affinity Gel beads (Millipore, P6611; approximately 0.5mL beads for 100mL culture in auto-induction media) at room temperature for 1h and rotated gently. Samples were loaded onto Proteus '1-Step Batch' Midi Spin Columns (Neo Biotech, NB-45-00058-2) and washed twice with 100mM NaCl, 10mM imidazole in PBS pH8. BG4 antibody was eluted with 700-1000μL elution buffer (250mM imidazole in PBS pH8.0) and dialyzed twice at 4°C overnight against filtered PBS using Maxi GeBaFlex dialysis tubes (Generon, D045). Aliquots were snap-frozen and stored at -80°C. The size and concentration of the BG4 antibody were determined by PAGE analysis. Affinity and specificity were determined by standard ELISA methods[2].

Transposome preparation

As the BG4 scFv construct is FLAG-tagged, we first removed the three tandem FLAG-tag motifs from the 3XFlag-pA-Tn5-Fl plasmid (a kind gift from Steven Henikoff. Addgene plasmid #124601; http://n2t.net/addgene:124601; RRID:Addgene_124601). 1μg of 3XFlag-pA-Tn5-Fl plasmid DNA was digested with NcoI-HF (NEB, R3193S) and HindIII-HF (NEB, R3104S) in 1X CutSmart Buffer (NEB, B7204S) at 37°C for 30min. Following incubation at 80°C for 20min, the digested plasmid was dephosphorylated with rSAP (NEB, M0371S) in 1X CutSmart Buffer at 37°C for 30min. After inactivating the enzyme at 65°C for 5min, the digested and dephosphorylated plasmid was purified from a 1% agarose gel using QIAquick Gel Extraction Kit (QIAGEN, 28704) according to the manufacturer's instructions. The digested plasmid was then ligated to the following annealed and phosphorylated oligonucleotides (All oligonucleotides were purchased from Sigma):

5'-CATGGGTATGACCATGATTACGCCA-3'

5’-AGCTTGGCGTAATCATGGTCATACC-3’

100µM of oligonucleotides were phosphorylated using T4 PNK (NEB, M0201S) in 1X T4 DNA ligase buffer (NEB, B0202A) at 37°C for 30min. Annealing was performed by heating to 95°C for 5min, followed by cooling at 2°C per min for 38 cycles. ~ 50ng of digested 3XFlag-pA-Tn5-Fl plasmid and ~ 506.5pg of annealed oligonucleotides were ligated using T4 DNA ligase (NEB, M0202S) in 1X T4 DNA ligase buffer (NEB, B0202A) at 25°C for 10min. The ligated plasmid was transformed to NEB® 5-alpha competent *E. coli* (NEB, C2987H) following the manufacturer’s protocol. Colonies were incubated in LB media, 100µg/mL ampicillin and plasmid DNA were extracted using QIAPrep Miniprep Kit (QIAGEN, 27106). Plasmids were screened for insertion by restriction enzyme digestion using BamHI-HF (NEB, R3136S) and BspDI (NEB, R0557S) and confirmed by Sanger sequencing (Eurofins).

The pA-Tn5 fusion plasmid lacking FLAG tags was transformed to C3013I cells (NEB) and expressed essentially as described by Kaya-Okur *et al.*[3] Bacteria were inoculated into 15mL LB broth,100µg/mL carbenicillin (Invitrogen, 10177012) and shaken at 250rpm at 37°C for 4h before adding to 1L of pre-warmed LB broth,100µg/mL carbenicillin and culturing. At OD_{600} ~0.7, the culture was cooled to 4°C for 1h in the cold room before addition of 250µl 1M IPTG (Thermo Scientific, R0392). Culturing was then continued at 200rpm, 18°C overnight. Bacterial pellets were collected by centrifugation at 9,500xg at 4°C for 1h and resuspended in 100mL chilled HEGX+PI (0.02M HEPES-KOH pH7.2, 1M NaCl, 0.001M EDTA, 10% glycerol, 0.2% Triton X-100, water, Roche Complete Protease Inhibitor EDTA-free tablet) in the cold room. Bacteria were lysed by sonication on ice using a Fisher Probe Sonicator (Fisher Scientific, FB705) with the following settings: Amp 20, Process time 00:04:00 (= eight cycles), Pulse ON 30 sec, Pulse OFF 1min. After eight cycles, the ice was

replaced and the sonication process was repeated. 100mL of lysed bacterial resuspension was centrifuged at 16,000xg at 4°C for 30min and supernatant harvested. In the cold room, 2.5mL fully resuspended chitin resin (NEB, S6651S) was added to five Econo-Pac Chromatography Columns (BIORAD, 732-1010) and washed in 20mL HEGX+PI. The bacterial supernatant was added, and the column sealed before incubating overnight at 4°C on a nutator. The next day, the unbound fraction was removed and the resin was washed twice with 20mL HEGX+PI. 6mL of HEGX,100mM DTT was added and the column sealed again and incubated at 4°C on a nutator for 48h. The eluate was collected and added to a Slide-A-Lyzer 10K MWCO dialysis cassette (Thermo Scientific, PI66830) and dialyzed against 2.5L 2X Tn5 dialysis buffer (0.1M HEPES-KOH pH7.2, 0.2M NaCl, 0.2mM EDTA, 1.7mM DTT, 0.2% Triton X-100, 20% glycerol, water), 1.7mM DTT. After 1.5h, the cassette was placed into fresh 2.5L 2X Tn5 dialysis buffer, 1.7mM DTT and incubated in the cold room overnight. 18mL of dialyzed protein was recovered and concentrated by centrifugation at 2,880xg at 4°C for ~1h using two Amicon Ultra-15 Centrifugal Filter Units (Merck, UFC901008). 4.3mL of concentrated protein was mixed with 4.3mL 80% glycerol and stored at -20°C. The size and concentration of purified pA-Tn5 fusion protein was measured using reducing PAGE on 4-12% Bis-Tris NUPAGE gels (Invitrogen, NP0321) according to the manufacturer with BSA (Pierce, 23209) as standards.

The transposome was then generated by incubating annealed MEDS oligonucleotides[4] with the pA-Tn5 fusion protein as described by Kaya-Okur *et al.*[3]. 200µL of 200µM Tn5MErev (5'-[phos]CTGTCTCTTATACACATCT-3') was annealed with 200µL of 200µM Tn5ME-A (5'- TCGTCGGCAGCGTCAGATGTGTATAAGAGACAG-3') or 200µL of 200µM Tn5ME-B (5'- GTCTCGTGGGCTCGGAGATGTGTATAAGAGACAG-3') in annealing buffer (10mM Tris-HCl pH8.0, 50mM NaCl, 1mM EDTA pH8.0 in water). 8µL of 100µM

pre-annealed MErev/ME-A and 8μL of 100μM pre-annealed MErev/ME-B were mixed with 100μL pA-Tn5 fusion protein incubated on a rotator for 1h at room temperature, and stored at -20°C.

Bulk G4-CUT&Tag

The CUT&Tag protocol described in Kaya-Okur *et al.*[3] for multi-cell (bulk) samples was adapted for G4 DNA secondary structures.

Cell preparation. U2OS cells were detached using accutase (Gibco, A1110501). Detached U2OS and K562 suspension cells were harvested by centrifugation and 100,000 cells per reaction were resuspended in 0.1% formaldehyde in PBS (Thermo Scientific, 28906) for 2min at room temperature, followed by quenching with glycine to a final concentration of 0.075M. Fixed cells were centrifuged at 1,300xg at 4°C for 4min and resuspended in wash buffer (20mM HEPES pH7.5, 150mM KCl, 0.5mM spermidine (Sigma, S0266)) in nuclease-free water with a Roche Complete Protease Inhibitor EDTA-free tablet (Sigma, 11873580001) at 1000 cells/μL. For experiments comparing different cell numbers, cell concentration was measured again at this point.

Bead capture. For seven samples, 70μL concanavalin A beads (Bangs Labs, BP531) were washed twice in 1-1.5mL binding buffer (20mM HEPES pH7.5, 10mM KCl, 1mM $CaCl_2$, 1mM $MnCl_2$ in nuclease-free water) and resuspended in 70μL binding buffer. 100μL of cell suspension was incubated with 10μL pre-washed concanavalin A beads in 1.5mL tube at 25°C for 10min at 600rpm. Cells bound to beads were washed twice in wash buffer using a magnetic rack (Invitrogen, 12321D) to capture the beads, before resuspending in 50μL antibody buffer (2mM EDTA, 0.1% BSA (Sigma, A8577), 0.05% digitonin (EMD Millipore, 300410) in wash buffer).

Tagmentation. 4µL of 5.4µM BG4 scFv was added to each sample and incubated at 25°C for 2h at 600rpm. Cells were washed twice with 100µL dig-wash buffer (0.05% digitonin in wash buffer) and resuspended in 50µL dig-wash buffer. 2µL of rabbit anti-FLAG antibody (Cell Signaling Technology Cat# 2368, RRID:AB_2217020) was then added and incubated at 25°C for 1h at 600rpm, followed by three washes in 500µL dig-wash buffer and resuspension in 50µL dig-wash buffer. Next, 0.5µL of anti-rabbit antibody (Antibodies-Online Cat# ABIN101961, RRID:AB_10775589) was added and incubated at 25°C for 1h at 600rpm. After 3 washes with 500µL dig-wash buffer, cells were incubated in 1:250 pA-Tn5 in 50µL Dig-300 buffer (20mM HEPES pH7.5, 300mM KCl, 0.5mM spermidine, 0.01% digitonin in nuclease-free water with a Roche Complete Protease Inhibitor EDTA-free tablet) at 25°C for 1h at 600rpm. Cells were then washed 3X in 500µL Dig-300 buffer before incubation in 300µL tagmentation buffer (10mM $MgCl_2$ in Dig-300 buffer) at 37°C for 1h at 600rpm.

DNA extraction. After tagmentation, cells were washed twice with 500µL TAPS wash buffer (10mM TAPS (Alfa Aesar, J63268.AE), 0.2mM EDTA in nuclease-free water). 100µL of extraction buffer (0.5mg/mL proteinase K (Thermo Scientific, EO0491), 0.5% SDS in 10mM Tris-HCl pH8.0) was added, vortexed and incubated at 55°C for 1h at 800rpm.100µL phenol-chloroform-isoamyl alcohol (Invitrogen, 15593049) was added and mixed. The mixture was transferred to MaXtract High Density Tube (QIAGEN, 129046) and centrifuged at room temperature for 3min at 16,000xg. 100µL chloroform was added to the top aqueous phase, mixed by inverting the phase-lock tubes ten times and then centrifuged at room temperature for 3min at 16,000xg. The top aqueous layer was transferred to a 1.5mL DNA Lo-bind tube (Eppendorf, 022431021). 4µL 5M NaCl and 200µL cold 100% ethanol were added, mixed and incubated at -20°C overnight. Samples were then centrifuged at 4°C for 30min at 21,130xg. The supernatant was carefully poured off and the DNA pellet rinsed with 1mL cold 100% ethanol and centrifuged at 4°C for 2min at 21,130xg. After pouring off the wash and

draining the residual liquid with paper towel, the pellet was left to air-dry. The pellet was finally redissolved in 25µL elution buffer 1 (10mM Tris-HCl pH8, 1mM EDTA, 1/400 RNAseA (Thermo Scientific, EN0531) in nuclease-free water) by vortexing and incubating at 37°C for 10min at 800rpm.

Library preparation. In a 0.2mL PCR tube, 25µL NEBNext HiFi 2x PCR master mix (NEB, M0541), 2µL of 10µM Ad1[4], 2µL of 10µM Ad2[4] and 21µL tagmented DNA were added and subjected to PCR (72°C for 5 min, 98°C for 30s, followed by 10 cycles of 98°C for 10s and 63°C for 10s, and one cycle of 72°C for 1 minute). Libraries were purified using 1.3X ratio (65µL) Ampure XP beads (Beckman Coulter, A63882). After 10min room temperature incubation, bead-bound DNA was washed twice with 80% ethanol and libraries eluted with 25µL 10mM Tris-HCl for 5min at room temperature.

Library sequencing. Library size and concentration were measured using a TapeStation HSD1000 ScreenTape. Libraries were balanced and pooled for size selection using Ampure XP beads. 0.4X ratio of Ampure XP beads were added to pooled libraries and the supernatant transferred to a new tube after 15min at room temperature. 1.4X ratio of Ampure XP beads were then added to the supernatant and incubated for 15min at room temperature. Beads were washed twice with 80% ethanol and libraires eluted in 40µL 10mM Tris-HCl. Libraries were sequenced on a NextSeq 500 sequencer (Illumina) with a paired-end format of 36 bp x 2 using the High Output kit (Illumina, FC-404-2005).

Bulk G4-CUT&Tag data processing

Sequencing quality was checked using FastQC. Bases with a quality score below 20 were trimmed from both reads using cutadapt (cutadapt -q 20). Resulting reads were aligned to hg38 with bwa mem. Aligned reads not overlapping the ENCODE blacklist were saved into

bam files (samtools view -Sb -F780 -q 10). Duplicate reads were removed using Picard (Picard MarkDuplicates) and deduplicated bam files sorted and indexed.

Peak calling. Deduplicated bam files were transformed into bedpe files (bedtools bamtobed -bedpe) and then only fragments with size below 1000bp (awk '{if ($1==$4 && $6-$2 < 1000) print $0}') were retained. Next, coverage of <1000bp fragments across human genome was computed using bedtools genomecov and reported in bedgraph format. SEACR was then used to identify regions of local enrichments. SEACR stringent search was performed to select top 5% peaks based on total signal within peaks (SEACR_1.3 $bdg_1000 0.05 non stringent). Peaks with a minimum coverage of eight reads were kept for further analysis.

Consensus regions and reference comparisons. Peaks obtained from individual libraries were combined together if replicates were available. Regions observed in both technical replicates (n=2) were selected as representative of the biological sample (multi2). When multiple biological replicates (i.e. different passages) were available, multi2 regions observed in a minimum two were used to generate consensus regions (bio). Regions of interest were compared to OQs[5] and previously reported G4 maps[6,7] by calculating the percentage of overlaps.

Assessment of reproducibility and sensitivity. To assess reproducibility and sensitivity, for different cell types, replicates and input cell number, the signal normalised to library size was computed for all libraries. First a super-set of consensus regions across all libraries was obtained by merging and sorting all consensuses across various cell types/assay conditions. Signal in those regions was then computed (bedtools coverage) and normalised to the total library size (reads per million, CPM). All libraries CPM were combined together into a single matrix, z-score was calculated and rescaled between 0 and 1. The resulting z-scores, where 0 indicate low and 1 high values, were used to perform unsupervised hierarchical clustering and to compute Spearman's rank correlations coefficients (r_s).

Supplementary Figures

a

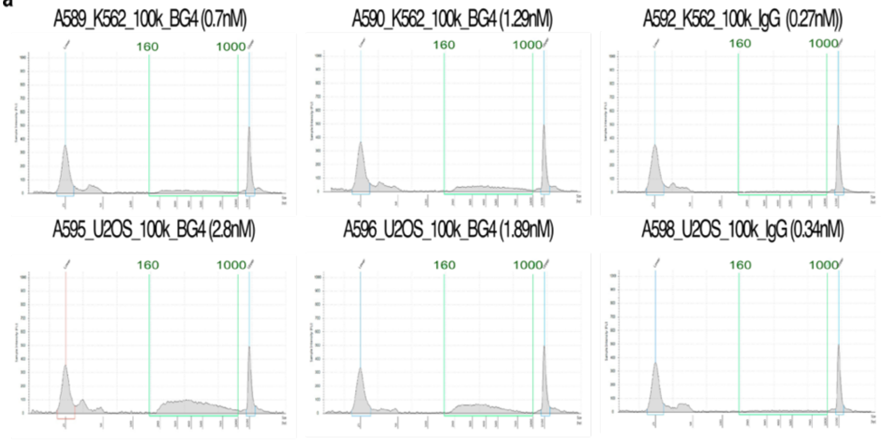

b

id	cell	ab1	rep	tech	lib pooled (μL)	mapped	mapped_no_dup	perc_dup
A589	K562	bg4	rep1	tec1	5	20,895,180	8,987,804	56.99
A590	K562	bg4	rep1	tec2	3	22,256,368	10,427,214	53.15
A592	K562	None	rep1	tecx	14	30,743,990	3,811,788	87.6
A603	K562	bg4	rep2	tec1	6	33,201,904	16,254,436	51.04
A604	K562	bg4	rep2	tec2	4	39,908,396	15,048,608	62.29
A606	K562	None	rep2	tecx	13	32,712,556	3,526,386	89.22
A628	K562	bg4	rep3	tec1	1.5	44,374,228	19,122,474	56.91
A629	K562	bg4	rep3	tec2	1.9	33,840,458	14,752,868	56.4
A630	K562	None	rep3	tecx	5.7	43,295,120	2,865,564	93.38
A595	U2OS	bg4	rep1	tec1	1	17,726,964	12,277,176	30.74
A596	U2OS	bg4	rep1	tec2	2	24,482,550	16,030,870	34.52
A598	U2OS	None	rep1	tecx	11	17,415,784	3,217,264	81.53
A609	U2OS	bg4	rep2	tec1	2	24,295,626	13,588,332	44.07
A610	U2OS	bg4	rep2	tec2	2	22,881,106	12,590,316	44.98
A611	U2OS	None	rep2	tecx	20	34,999,250	3,577,146	89.78
A632	U2OS	bg4	rep3	tec1	0.7	32,453,716	18,913,320	41.72
A633	U2OS	bg4	rep3	tec2	2	30,511,232	16,803,610	44.93
A634	U2OS	None	rep3	tecx	10.2	53,757,750	3,179,388	94.09

Supplementary Fig. S1 | Comparison of G4-CUT&Tag libraries relative to negative controls. For each biological replicate, a negative control was included with the same number of starting cells and PCR cycles without adding the BG4 scFv primary antibody. **a,** Size profiles and library quantification measured using a TapeStation with High Sensitivity D1000 ScreenTape (Agilent). x-axis: library size from 25bp to 1,500bp; y-axis: sample intensity [FU]; green lines: indicate libraries ranged from 160bp to 1,000 bp. **b,** Basic mapping statistics of K562 and U2OS G4-CUT&Tag and control libraries: the table reports the primary antibody used (ab1), three biological replicates (rep1, rep2, rep3) with two technical replicates (tec1, tec2) and one negative control (tecx) for K562 and U2OS cells, total number of mapped reads (mapped), total number of mapped reads after duplicate removal (mapped_no_dup) and the corresponding duplication rate (perc_dup).

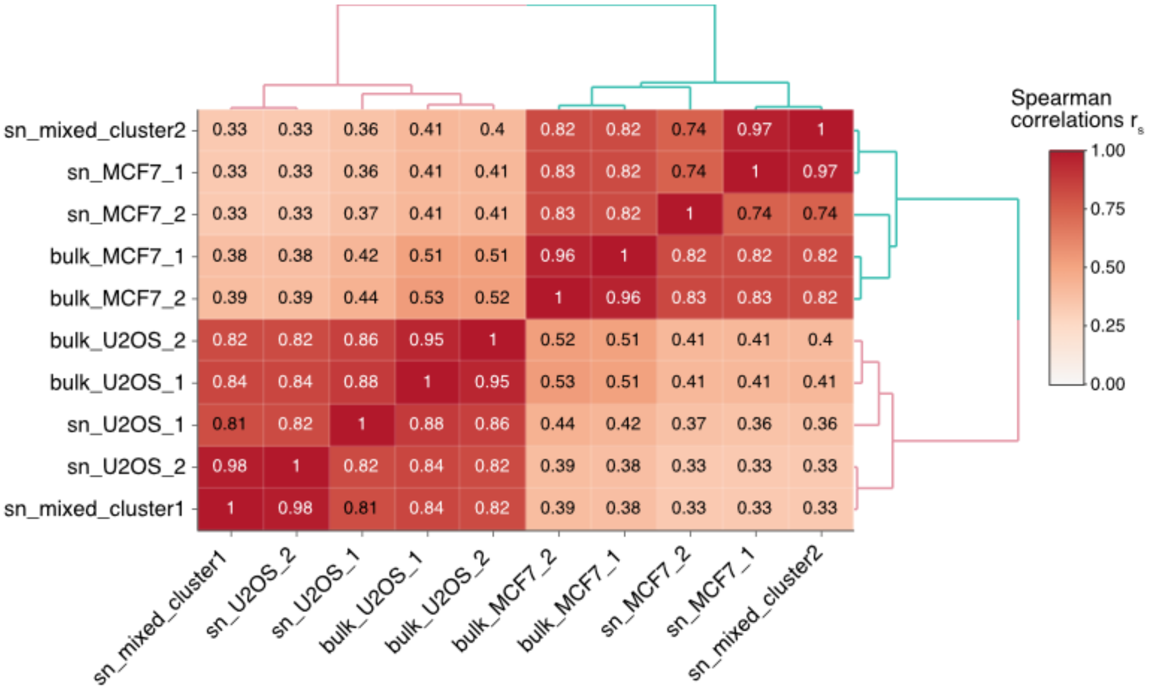

Supplementary Fig. S2 | Single-nuclei G4-CUT&Tag can identify separate cell types in a mixed population. Hierarchical clustering of the Spearman correlation matrix of single-nuclei and bulk G4-CUT&Tag replicates of U2OS and MCF7 cells. The column and row names indicate individual single nuclei (sn_) and bulk (bulk_) replicates (U2OS_1, U2OS_2, MCF7_1, MCF7_2) and the experiment where two cell lines have been mixed and then identified as different clusters (sn_mixed_cluster1, sn_mixed_cluster2). Spearman correlations between samples were computed based on the normalised read coverages at SEACR G4 peaks identified after sequencing bulk data.

a

	Min.	1st Qu.	Median	Mean	3rd Qu.	Max.	Number of regions
commonRegions_Ncells_U2OS	1	17	25	28	35	348	491
commonRegions_Ncells_MCF7	1	12	19	24	28	289	491
specifRegions_Ncells_U2OS	1	1	2	7	10	87	9810
specifRegions_Ncells_MCF7	1	1	2	6	6	107	2361

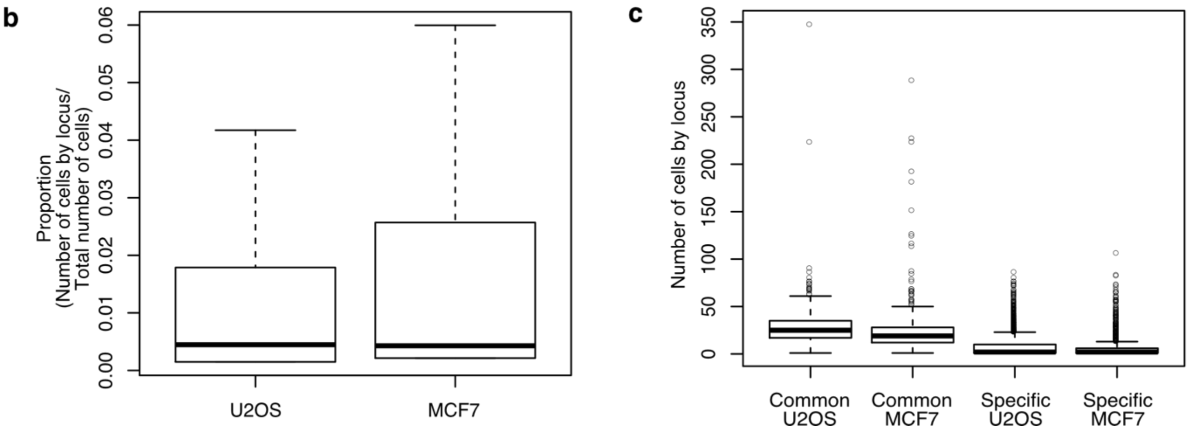

Supplementary Fig. S3 | Subtypes of G4s in snG4-CUT&Tag data of mixed U2OS and MCF7 cells. a, Statistics on the number of cells supporting G4 peaks (based on peaks called by MACS2 in aggregated data from each cluster) in two cell lines. **b,** Boxplot showing the distribution of the proportion of cells at G4 peaks in both cell lines. The center line indicates median, the lower and upper lines of the box correspond to the first and third quartiles, and the upper and lower whiskers extend to the maximum value no further than 1.5x interquartile range. **c,** Boxplot showing the distribution of the number of cells across G4 peaks: common G4 peaks (regions observed in both U2OS and MCF7 cells), G4 peaks specific to U2OS cells and G4 peaks specific to MCF7 cells. The center line indicates median, the lower and upper lines of the box limits correspond to the first and third quartiles, and the upper and lower whiskers extend to the maximum value no further than 1.5x interquartile range, with points indicate potential outliers.

References

1 Biffi, G., Tannahill, D., McCafferty, J. & Balasubramanian, S. Quantitative visualization of DNA G-quadruplex structures in human cells. *Nat Chem* **5**, 182-186, doi:10.1038/nchem.1548 (2013).

2 Hänsel-Hertsch, R., Spiegel, J., Marsico, G., Tannahill, D. & Balasubramanian, S. Genome-wide mapping of endogenous G-quadruplex DNA structures by chromatin immunoprecipitation and high-throughput sequencing. *Nature Protocols* **13**, 551-564, doi:10.1038/nprot.2017.150 (2018).

3 Kaya-Okur, H. S. *et al.* CUT&Tag for efficient epigenomic profiling of small samples and single cells. *Nat Commun* **10**, 1930, doi:10.1038/s41467-019-09982-5 (2019).

4 Buenrostro, J. D. *et al.* Single-cell chromatin accessibility reveals principles of regulatory variation. *Nature* **523**, 486-490, doi:10.1038/nature14590 (2015).

5 Chambers, V. S. *et al.* High-throughput sequencing of DNA G-quadruplex structures in the human genome. *Nat Biotechnol* **33**, 877-881, doi:10.1038/nbt.3295 (2015).

6 Shen, J. *et al.* Promoter G-quadruplex folding precedes transcription and is controlled by chromatin. *Genome Biol* **22**, 143, doi:10.1186/s13059-021-02346-7 (2021).

7 Spiegel, J. *et al.* G-quadruplexes are transcription factor binding hubs in human chromatin. *Genome Biol* **22**, 117, doi:10.1186/s13059-021-02324-z (2021).
